# Micro-RNA-Regulated *SQUAMOSA-PROMOTER BINDING PROTEIN-LIKE* (*SPL*) Gene Expression and Cytokinin Accumulation Distinguish Early-Developing Male and Female Inflorescences in Oil Palm (*Elaeis guineensis*)

**DOI:** 10.3390/plants11050685

**Published:** 2022-03-02

**Authors:** James W. Tregear, Frédérique Richaud, Myriam Collin, Jennifer Esbelin, Hugues Parrinello, Benoît Cochard, Leifi Nodichao, Fabienne Morcillo, Hélène Adam, Stefan Jouannic

**Affiliations:** 1 DIADE, University of Montpellier, CIRAD, IRD, 34394 Montpellier, France; myriam.collin@ird.fr (M.C.); j.esbelin@gmail.com (J.E.); fabienne.morcillo@ird.fr (F.M.); helene.adam@ird.fr (H.A.); stephane.jouannic@ird.fr (S.J.); 2 CIRAD, UMR AGAP, 34398 Montpellier, France; frederique.richaud@cirad.fr; 3 AGAP, University of Montpellier, CIRAD, INRAE, Institut Agro, 34398 Montpellier, France; 4 MGX-Montpellier GenomiX, University of Montpellier, CNRS, INSERM, 34094 Montpellier, France; hugues.parrinello@mgx.cnrs.fr; 5 PalmElit SAS, 34980 Montferrier sur Lez, France; benoit.cochard@palmelit.com; 6 INRAB, Station INRAB CRA-PP, Pobè BP 01, Benin; anleifi@yahoo.com; 7 CIRAD, UMR DIADE, 34394 Montpellier, France

**Keywords:** inflorescence, branching, sexual differentiation, oil palm, miRNA, SQUAMOSA PROMOTER-BINDING PROTEIN

## Abstract

Sexual differentiation of inflorescences and flowers is important for reproduction and affects crop plant productivity. We report here on a molecular study of the process of sexual differentiation in the immature inflorescence of oil palm (*Elaeis guineensis*). This species is monoecious and exhibits gender diphasy, producing male and female inflorescences separately on the same plant in alternation. Three main approaches were used: small RNA-seq to characterise and study the expression of miRNA genes; RNA-seq to monitor mRNA accumulation patterns; hormone quantification to assess the role of cytokinins and auxins in inflorescence differentiation. Our study allowed the characterisation of 30 previously unreported palm *MIRNA* genes. In differential gene and miRNA expression studies, we identified a number of key developmental genes and miRNA-mRNA target modules previously described in relation to their developmental regulatory role in the cereal panicle, notably the *miR156/529/535-SQUAMOSA PROMOTER-BINDING PROTEIN-LIKE* (*SPL*) gene regulatory module. Gene enrichment analysis highlighted the importance of hormone-related genes, and this observation was corroborated by the detection of much higher levels of cytokinins in the female inflorescence. Our data illustrate the importance of branching regulation within the developmental window studied, during which the female inflorescence, unlike its male counterpart, produces flower clusters on new successive axes by sympodial growth.

## 1. Introduction

Flowering plants have evolved a wide range of architectural features to ensure their reproductive success, including the inflorescence, which carries the flowers which later form fruits or grains. Inflorescence architecture is very diverse in flowering plants and provides a means to optimize important reproductive factors such as pollination, seed dispersal, and the separation of sexes to promote outbreeding. In crop plants, inflorescence structure is an agronomically important character and often affects yield, which depends on the number of flowers available for fertilization. Much research into the determination of inflorescence architecture has focused on cereals due to their great importance to world agriculture and human nutrition [1], particularly rice and maize [2], whilst a range of eudicot species such as *Arabidopsis thaliana* have also been studied [3].

One important feature of the pathways controlling plant inflorescence structure is the involvement of post-transcriptional regulation mediated by micro-RNAs (miRNAs). These molecules are 21–24 nucleotide (nt) noncoding RNAs that target specific mRNAs for cleavage or translational repression, thereby modulating expression levels and participating in a wide range of physiological and developmental processes in plants and animals [4]. The biogenesis of miRNAs in plants has been extensively described [5,6]. These molecules target specific messenger RNAs (mRNAs) for cleavage or translational repression [7], cleavage appearing to be the most common mechanism in plants. In the context of inflorescence development, two miRNA types, in particular, have been noted to play key roles in monocots. Firstly, *miR172*, encoded by the *TASSELSEED4* gene in maize, has been shown to post-transcriptionally regulate expression of the *INDETERMINATE SPIKELET* gene orthologous to the *A. thaliana APETALA2* gene, *tasselseed4* mutants displaying reduced inflorescence branching with additional sex-dependent effects on the male and female inflorescences [8]. Secondly, the three different miRNAs that target transcripts of *SQUAMOSA PROMOTER BINDING-LIKE* (*SPL*) genes have been found to play roles in inflorescence development. The three plant miRNA classes in question, *miR156*, *miR529*, and *miR535*, can be considered a superfamily on account of their high sequence similarity [9]. Through their involvement in regulating *SPL* gene expression, *MIR156*/*MIR529*/*MIR535* genes influence many aspects of plant growth and development, including flowering. In the case of *miR156*, a role in the determination of inflorescence branching was elucidated through studies of a rice *SPL* gene identified through the quantitative trait loci *WEALTHY FARMER*’*S PANICLE* [10] and *IDEAL PLANT ARCHITECTURE* (*IPA*) [11]. The latter encodes the OsSPL14 protein (SQUAMOSA PROMOTER BINDING PROTEIN-LIKE 14, also known as IPA1), a higher expression of which promotes panicle branching and therefore higher grain yield. Mutation of the miRNA-binding site in the *IPA1* mRNA, rendering it resistant to *miR156*, results in an overexpression phenotype. As mentioned previously, like *miR156*, *miR529* and *miR535* are also able to target mRNAs of the *SPL* family. Sequence differences between *miR156* and *miR529* relate to their mismatch-sensitive regions, which determine target recognition. On the basis of rice studies, Yan et al. [12] inferred that *miR529a* might control growth and development by regulating SPL target genes at different stages compared with *miR156* and suggested that *miR529a* might play a dominant role in the regulation of target genes during early panicle development. As regards *miR535*, overexpression studies in rice revealed a role for this miRNA in the regulation of panicle architecture, plant height and grain length [13]. *OsmiR535* is highly expressed in panicles and may be induced in response to certain abiotic stresses [14].

Inflorescence branching complexity varies in palms (monocot family Arecaceae), with branching orders ranging from zero to six [15]. Sexual expression (spatial and temporal separation of male and female reproductive organs) is also highly variable in this group [16]. African oil palm (*Elaeis guineensis*), the world’s largest source of vegetable oil, is monoecious but produces functionally unisexual male and female inflorescences on the same plant. Primary inflorescence branches found on both are known as rachillae. A histomorphological study of *E. guineensis* inflorescence and flower development was carried out previously [17]. In favorable conditions, a new inflorescence is produced every two weeks in each successive leaf axil, the youngest expanding leaf defined as leaf +1. Male and female inflorescences are produced in alternation, a phenomenon known as gender diphasy. The production of male inflorescences is known to be favored by conditions of water stress, although in extreme conditions, inflorescences may be aborted. Over two years elapse between inflorescence initiation and flower maturity. Inflorescence sex is determined 7.5–10 months after initiation [18,19] when the first appearance of rachilla bracts (approximately leaf −3) defines the future number of secondary inflorescence axes (one per bract). Male inflorescences carry more primary branches than their female counterparts, but a clear distinction of the two sexes is only possible 6–12 months later, when bract density is higher on the male rachillae than on the female rachillae [20]. The male rachillae produce single isolated male flowers, whereas the female rachillae produce floral triads. The latter are sympodially branched clusters consisting of two successively initiated abortive male flowers followed by a fertile female flower. Branching on the oil palm inflorescence is, therefore, both qualitatively and quantitatively sex dependent.

Our study was aimed at understanding how prereproductive sexual differentiation occurs in oil palm inflorescences. We characterized miRNAs and studied sex-related differential gene expression in male and female inflorescences, which were also subjected to hormone analyses. Our data provide an insight into the molecular processes that regulate inflorescence sexual differentiation in this species.

## 2. Results

### 2.1. Characterisation of miRNAs Accumulating in the Immature Oil Palm Inflorescence

**Figure 1:**
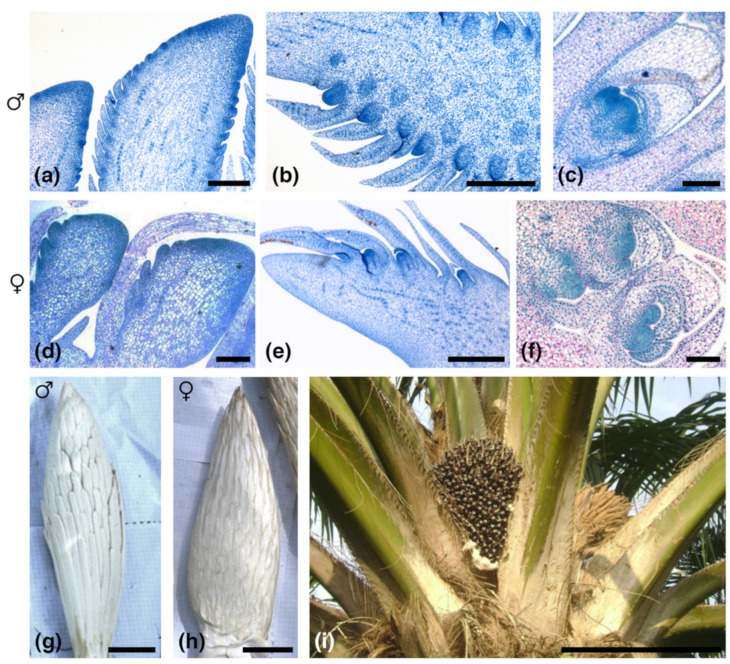
Stages of oil palm inflorescence development; microscopic views in panels (**a**–**f**) and macroscopic views in panels (**g**–**i**). (**a**) Longitudinal section of male rachilla (leaf +5) showing young floral bracts; (**b**) Longitudinal section of male rachilla (leaf +12) showing flower primordia; (**c**) Longitudinal section of male rachilla (leaf +15) showing early differentiating male flower; (**d**) Longitudinal section of female rachilla (leaf 0) showing floral triad bracts; (**e**) Longitudinal section of female rachilla (leaf +10) showing floral triad initiation; (**f**) Longitudinal section of female rachilla (leaf +14) showing floral triad development; (**g**) Nearly mature male inflorescence (leaf +17); (**h**) nearly mature female inflorescence (leaf +16); (**i**) crown of adult palm showing infructescence and senescent male inflorescence. Scale bars: (**a**,**c**,**d**,**f**), 0.1 mm; (**b**), 0.5 mm; (**e**), 1 mm; (**g**,**h**), 5 cm; (**i**), 50 cm.

Small RNA-seq data, obtained from each of the three male and three female immature inflorescence samples (developmental stages indicated in Figure S1 and Figure 1), were processed and mapped to the oil palm reference genome EG5 in order to detect and measure the abundance of known and novel *MIRNA* genes expressed in the tissues of interest.

**Figure 2:**
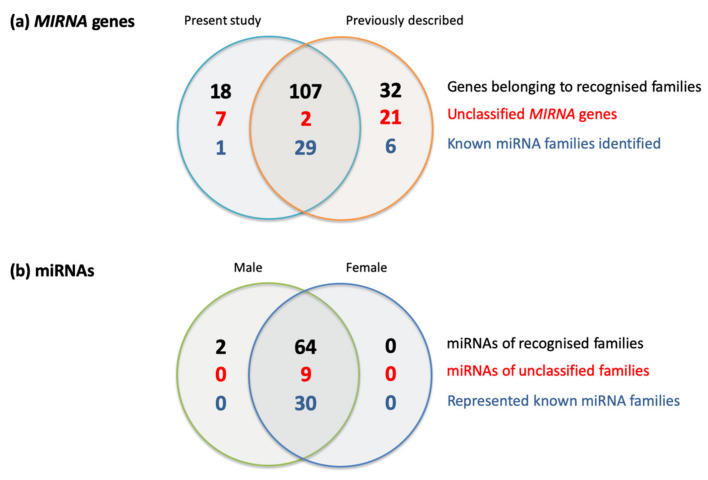
Venn diagrams summarizing oil palm miRNA data from the present study along with pre-existing (sRNAanno database) data.

A total of 134 miRNA-encoding genes were identified in the present dataset (Figure 2) [21], representing 30 recognized *MIRNA* gene families. Database comparisons revealed that 109 of the 134 genes had been previously identified in oil palm (http://www.plantsrnas.org accessed on 5 January 2022), whereas the remaining 25 genes were previously unreported in this species. Compared with database sequences, only one gene in the present dataset (encoding *miR398*) belonged to a recognized family that was previously unreported in oil palm. Otherwise, 18 of the previously unreported genes were members of known miRNA families, while the other 7 genes were classified as novel on the basis of their mature miRNA sequences being unrelated to any catalogued group. The deduced precursor and mature sequences associated with each gene, along with their corresponding genomic locations, are shown in Table S1. In some cases, a small difference in the position of the mature miRNA sequence was observed compared with the earlier annotation (15 instances noted). Similarly, in 10 cases, an inversion was seen between the miRNA sequence designated as the “mature” form in the earlier annotation and that designated as the “star” form. Since the present annotations were based on sRNA abundance in the immature oil palm inflorescence, we used these as the basis for subsequent analyses. The accumulation profile across the three male and three female inflorescence samples of each identified mature miRNA form, expressed as normalized per million values, is shown in Table S2, along with their corresponding target mRNA types (in the case of known miRNA families) or potential mRNA targets (identified using the psRNATarget server in the case of unclassified miRNAs; [22]). Only two miRNAs (*miR156d* and *miR319g*) showed sex-specific accumulation (male inflorescence only), but in both cases abundance was very low, and *miR319a* was only detected in one of the six samples. Applying an arbitrary threshold of 2-fold to the M/F ratio, we observed 19 miRNAs that were more abundant in the male samples and 8 miRNAs displaying female-enriched accumulation. Indicated in Table S2 are those miRNAs for which we were able to identify potential mRNA targets within the DEG sets using the psRNATarget server (see below). Although the majority of the oil palm *MIRNA* genes identified in this study were already catalogued in the sRNAanno database, nearly one-fifth of them (18.7%) are reported here for the first time, thus enriching the existing catalogue of *MIRNA* genes present in the oil palm genome.

### 2.2. Gene Expression Patterns Distinguish Early Developing Male and Female Oil Palm Inflorescences

Illumina mRNA-seq was performed on the same set of male and female inflorescence samples used for the miRNA analysis. A global gene expression heatmap analysis based on a set of 50 genes showing the most variable expression across all 8 samples revealed a clustering according to inflorescence sex (Figure S2). Analysis performed using the DESeq2 package, applying a Padjust threshold of 0.05, allowed the identification of 922 differentially expressed genes (DEGs), of which 286 were more highly expressed in the male inflorescence (“M_up” category, corresponding to a negative log_2_fold change value) and 636 were more highly expressed in the female inflorescence (“F_up” category, corresponding to a positive log_2_fold change value). The DEGs are listed in Table S3, along with their orthologs, where identified, in the model species *A. thaliana* and *Oryza sativa*.

### 2.3. Key Regulatory Genes Displaying Sex-Dependent Expression Are miRNA-Regulated

In order to evaluate the importance of miRNA-mediated regulation of gene expression in the early sexual differentiation of male and female inflorescences in oil palm, we performed database searches using the psRNATarget server (https://www.zhaolab.org/psRNATarget/ accessed on 5 January 2022) [22] to identify potential mRNA targets of each characterised miRNA that were also represented in either the M_up or F_up DEG sets. A summary of the information obtained is shown in Table 1, in which only miRNAs displaying a male/female differential accumulation profile greater than 2 or less than 0.5 are listed. Since plant miRNAs generally target specific mRNAs for post-transcriptional degradation, a reverse accumulation profile between miRNA and mRNA can provide a useful means to assess the likelihood of an in vivo interaction. In Table 1, those miRNAs that display a reverse accumulation profile compared to their putative target mRNAs are highlighted in blue.

Overall, it can be seen that the miRNAs with the most strongly sex-dependent accumulation patterns were generally those for which putative target mRNAs displayed a reversed profile. The most striking feature is the fact that the five miRNAs displaying the most strongly differential accumulation are all likely to target one or more of the three different *SQUAMOSA BINDING PROTEIN-LIKE* (*SPL*) genes found in the F_up DEG set. Indeed, all five miRNAs displayed a reversed accumulation profile compared with their likely targets, i.e., with higher abundance (at least 7-fold greater) in the female inflorescence. Three of the oil palm miRNAs thus identified belong to the *miR156* family, while the other two miRNAs belong to the *miR529* and *miR535* families, respectively. One of the potential mRNA targets of these miRNAs, which we designated as *EgSPL14-2* (from oil palm gene LOC105061255), is orthologous to that of the rice *IDEAL PLANT ARCHITECTURE1* (*IPA1*; LOC_Os08g39890) gene mentioned earlier [10,11]. Two other oil palm *SPL* genes, *EgSPL14-1* and *EgSPL14-3*, corresponding to loci LOC105050210 and LOC105050485, both identified as potentially miRNA-regulated and both displaying higher expression in the female inflorescence, were also identified. All three oil palm genes belong to the same *SPL* subfamily, as defined by Preston and Hileman [23] (Figure S3). After the miRNAs targeting *SPL* mRNAs, the most strongly differential miRNA was *miR5179*, for which the putative target is the oil palm MADS-box transcription factor gene *EgDEF1* orthologous to the class B *DEFICIENS* gene [24]. The only species for which *miR5179* targeting of *DEFICIENS* ortholog mRNAs has thus far been experimentally demonstrated is the orchid *Orchis italica* [25]; however, this interaction has also been inferred from sequence data from other monocot species such as *Cymbidium ensifolium* [26] and coconut [27]. Although *miR5179* is shown in the databases as also occurring in cereal and eudicot species, no experimentally validated targets appear to have been described outside monocots to date, raising the question that this miRNA/mRNA module might be monocot specific.

In addition to the aforementioned cases, another regulatory module of likely significance to inflorescence development that we observed was that of *AUXIN RESPONSE FACTOR* (*ARF*) gene transcripts (LOC105034684, LOC105055363) targeted by *miR167a* and *miR167b*. The involvement of auxin in pathways regulating inflorescence branching and development has been well established for many years [28], with ARF proteins playing an important role through their binding to the promoters of auxin-responsive genes [29]. Other *miRNAs* listed in Table 1 target additional gene families of interest to inflorescence development (*LACCASE* and *NAC* and families); however, it should be noted that in the latter cases, accumulation patterns of miRNAs were relatively similar between male and female inflorescences, and sometimes they were not reversed compared with the putative mRNA targets, thus implying that their involvement in post-transcriptional regulation was not a significant contributor to developmental regulation.

### 2.4. Gene Enrichment Analysis Reveals the Importance of Hormonal Regulation during Inflorescence Sexual Differentiation

**Figure 3:**
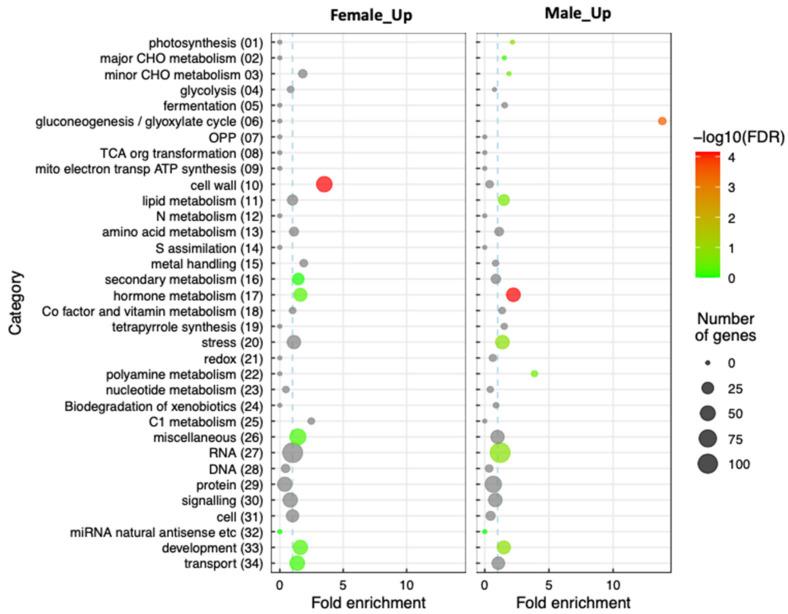
Enrichment of specific gene functional categories in the DEG sets as defined by Lohse et al. [30]. The color scale representing significance is based on log_10_ values calculated from the FDR-corrected *p* values in Table S5 using the R package of Bonnot et al. [31]. Fold enrichment calculated in Table S5 is represented on the x-axis (the vertical dotted blue line denoting an enrichment ratio of 1). Circle sizes represent the number of DEGs for each respective category as indicated. Grey circles indicate DEG set/functional category combinations with nonsignificant FDR-corrected *p* values.

In order to search as widely as possible for underlying factors that might be involved in the early sexual differentiation of the oil palm inflorescence, the Mercator/Mapman annotation system was used to assign functional categories to each of the DEGs. Next, the percentage representation of each gene functional category in the reference oil palm genome was compared with the corresponding percentage representations of genes of the same category within either the M_up or the F_up group, as detailed in Table S4. This allowed us to test for significant enrichment of each functional category within each DEG set. We detected three instances of significantly enriched gene categories (Fisher’s exact test with FDR cutoff < 0.05), as shown in Figure 3 and Table S5.

One instance of gene enrichment was detected in the F_up DEG set, namely the cell wall (category 10), and two different functional categories were highlighted in the M_up DEG set, namely gluconeogenesis/glyoxylate cycle (category 06) and hormone metabolism (category 17). The possible significances of these categories in oil palm inflorescence sexual differentiation are discussed later.

### 2.5. Analysis of Hormone Composition in Immature Male and Female Inflorescences

**Figure 4:**
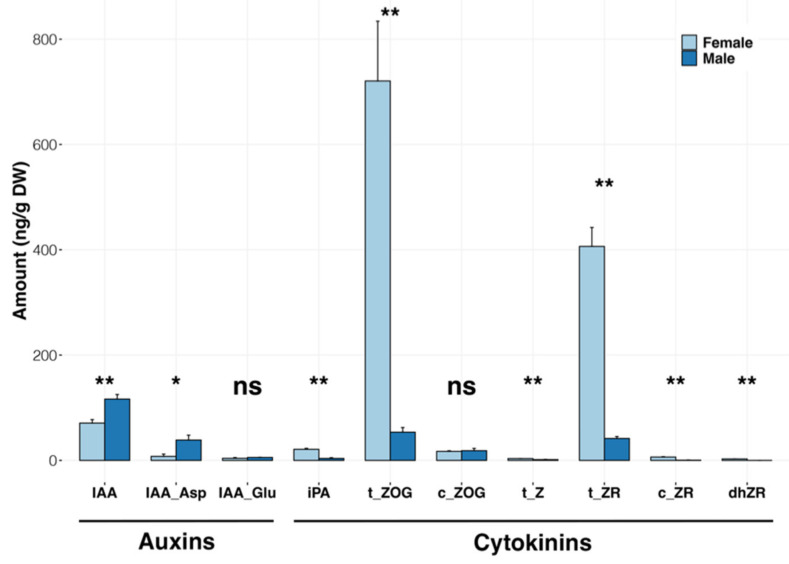
Analysis of the hormonal composition of oil palm male and female inflorescences. Abbreviations: IAA—indole-3-acetic acid; IAA-Asp—indole-3-acetyl-aspartate; IAA-Glu— indole-3-acetyl-glutamate; c-ZOG—cis-zeatin *O*—glucoside; c-ZR—cis-zeatin riboside; dhZR—dihydrozeatin riboside; iPA—isopentenyladenine; t-Z—trans-zeatin; t-ZOG—trans-zeatin *O*-glucoside; t-ZR— trans-zeatin riboside. Measurements are expressed as ng/g dry weight. Levels of significance calculated using the Wilcox method (R package rstatix) are indicated as follows: **—*p* value < 0.01; *—*p* value < 0.05; ns—not significant.

Since hormones play a central role in the development of plant reproductive structures, and in view of the various hormone-related DEGs and miRNAs characterized in earlier experiments, we analyzed the auxin and cytokinin contents of samples A04M, A05F, A06F, and A07M using the same tissue samples as were used for the transcriptomic analyses. Results were obtained for three different auxin molecules: indole-3-acetic acid (IAA); indole-3-acetyl-aspartate (IAA-Asp); and indole-3-acetyl-glutamate (IAA-Glu). For cytokinins, seven different forms were analyzed: cis-zeatin O-glucoside (c-ZOG); cis-zeatin riboside (c-ZR); dihydrozeatin riboside (dhZR); isopentenyladenine (iPA); trans-zeatin (t-Z); trans-zeatin O-glucoside (t-ZOG); and trans-zeatin riboside (t-ZR). The data obtained are shown in Figure 4 as histograms representing the numerical data in Table S6.

In the case of the auxin molecules, a significant difference between male and female inflorescences was observed for the molecule IAA, which accumulated at higher levels (M/F ratio 1.63, *p* < 0.01) in the male inflorescence, as well as for its conjugated form IAA-Asp (M/F ratio 5.07, *p* < 0.05). In the case of cytokinins, two forms predominated to a large degree, namely trans-zeatin *O*-glucoside (t-ZOG) and trans-zeatin riboside (t-ZR). In both cases, a much larger quantity was found to accumulate in the female inflorescence samples compared with male ones (t-ZOG F/M ratio 13.5, *p* < 0.01; t-ZR F/M ratio 9.8, *p* < 0.01). Moreover, four other more minor cytokinin forms were also found to be significantly more abundant (*p* < 0.01) in the female inflorescence, namely trans-zeatin (t-Z; F/M ratio 1.5), cis-zeatin riboside (c-ZR F/M ratio 16.12), isopentenyladenine (iPA F/M ratio 3.77), and dihydrozeatin riboside (dhZR detected only in the female inflorescence). Collectively, our biochemical data revealed a strong divergence between male and female inflorescences in the abundance of specific hormones, strongly suggesting a role for these molecules in the regulatory processes underlying inflorescence sexual differentiation.

## 3. Discussion

### 3.1. Post-Transcriptional miRNA-mRNA Modules Are Likely to Be Involved in Sex-Dependent Regulatory Processes in the Developing Oil Palm Inflorescence

In our study, five different miRNAs showing highly contrasting accumulation between male and female inflorescences belonged to families known to target *SPL* gene transcripts. In addition to three different sequence variants of *miR156* (*miR156a*, *miR156b*, and *miR156d*), sex-dependent accumulation was observed for *miR529a* and *miR535a*, the latter two miRNAs being generally more abundant than those of the *miR156* family. In the present study, all five oil palm miRNAs belonging to the *miR156*/*miR529*/*miR535* superfamily displayed a reversed accumulation profile compared with their likely targets, i.e., with higher abundance (at least 7-fold greater) in the female inflorescence. Their convergent expression patterns, along with the inverse profiles of several different target *SPL* genes transcripts (see below), point to a critical role for *SPL* gene activity as a key player in early oil palm inflorescence differentiation. The structural and functional evolution of the *SPL* gene family has been the focus of a large number of studies. On the basis of molecular phylogenies, Preston and Hileman [23] identified nine different clades of SPL proteins in plants, six of which correspond to miRNA-regulated groups. As regards the present study, the three differentially expressed oil palm *SPL* genes all encode proteins belonging to the miRNA-regulated clade IX (Figure S3). It is interesting to note that all members of this group, which also includes the rice IPA1 protein involved in inflorescence branching, are from monocot species. In maize, two closely related functionally redundant genes, named *UNBRANCHED2* (*UB2*) and *UNBRANCHED3* (*UB3*), are paralogous to the rice *IPA1* gene with which they share conserved functions. Recent studies have uncovered a number of signaling components that interact with *SPL* genes in the regulation of inflorescence development. In rice, the IPA1 transcription factor protein was shown to bind to the promoters of a number of key regulators of rice plant architecture, targets including the ortholog of the maize bHLH gene *TEOSINTE BRANCHED1* (*OsTB1*), the G protein γ subunit *DENSE AND ERECT PANICLE1* gene (*OsDEP1*), the cytokinin-activating *LONELY GUY* gene, the gibberellin response gene *SLENDER RICE1*, and the auxin transport gene *PIN-FORMED* [32]. *IPA1* activity has been shown to be negatively affected in panicles by IPA1 INTERACTING PROTEIN 1 (IPI1), a RING-finger E3 ligase [33], whereas in wheat and other cereal species, members of the PHYTOCHROME-INTERACTING FACTOR-LIKE (PIL) family of transcription factors have been shown to interact directly with SPLs, thereby repressing branching and tillering [34]. It is interesting to note that in the present study, an oil palm gene encoding a PIL type transcription factor was identified in the M_up DEG set, i.e., displaying an opposing expression profile compared with the *SPL* genes, as would be predicted from the aforementioned study.

As regards the other miRNAs displaying differential accumulation in relation to inflorescence sex, although no direct parallels can be drawn with data obtained from model plants, a functional significance can nevertheless be inferred in the case of *miR5179*, which putatively targets expression of the oil palm *DEFICIENS* ortholog *EgDEF1*, which displayed an inverse profile in our study. The *EgDEF1* gene encodes a MADS-box protein that regulates the identity of the corolla and androecium [35,36]. Although the inflorescence developmental stages represented in our sampling preceded the differentiation of the reproductive floral organs (i.e., the androecium and gynoecium), the activities of floral class B homeotic genes such as those related to DEFICIENS are also required for the development of the sterile whorls (sepals and petals). Our observation of expression of the *EgDEF1*/*miR5179* module in immature inflorescence tissues studied is, therefore, to be expected. Moreover, our observation that *EgDEF1* expression is higher in the female inflorescence samples than the male ones might be attributable to the different modes of development of the two structures, the former involving the sequential production of non-functional male flowers that abort before maturity, with the functional female flower the last to be initiated and to differentiate. Thus, the expression of floral homeotic genes is likely to cover a longer time period in the female inflorescence prior to anthesis, in contrast to the situation in the male inflorescence, which will require floral homeotic gene activity starting from a later stage of development, resulting in a weaker representation of *EgDEF1* transcripts in the pooled samples. Another differentially accumulating category of miRNAs, represented by *miR167a* and *miR167b*, which target *AUXIN RESPONSE FACTOR* (*ARF*) gene transcripts, may well reflect a differential modulation of auxin signalling pathways between the two inflorescence types linked to their contrasting branching morphologies, a key character affected by auxin [37,38,39]. The only female-enriched miRNA corresponding to a reversed profile DEG target was *miR397b*, putatively targeting transcripts of a laccase gene (LOC105036954). A link between inflorescence branching and overexpression of the miRNA-encoding gene *OSMIR397* has been observed in rice [40], in which a role for *miR397*-mediated laccase gene silencing was also observed in relation to domestication [41]; however, in the present study, it is difficult to conclude that this miRNA family plays a role in inflorescence differentiation given that *miR397b* and *miR397a* were found to display contrasting male- and female-enriched accumulation respectively, while both can potentially target transcripts of the LOC105036954 gene present in the M_up DEG set. The best-characterized role of the *miR397* family is its involvement in laccase-mediated lignin biosynthesis [32]; however, the functions of most laccase genes remain unknown [42].

Given its monoecious character, it is interesting to compare oil palm with maize, which also produces unisexual male and female inflorescences on the same plant and which, as mentioned above, employs a *miR172*/*APETALA2* module in the regulation of inflorescence sex determination and branching [8]. No evidence for the involvement of such a module in oil palm inflorescence architecture determination was obtained in the present study, suggesting that different mechanisms operate in these two species. Functional divergence between eudicots and grasses, in this respect, was noted previously [43]. It is nevertheless important to bear in mind when interpreting the miRNA/mRNA profiles that some biologically relevant patterns may be masked by differences in tissue-specific accumulation between the individual RNA species, with post-transcriptional interaction occurring only in certain specific histological regions. This may also explain why several miRNAs showed strongly differential accumulation between male and female inflorescences, but no potential mRNA targets were found to be represented in the DEG sets.

### 3.2. Global Expression and Biochemical Profiles Indicate a Key Role for Hormones in Oil Palm Inflorescence Sexual Differentiation

Our gene enrichment analyses performed on the M_up and F_up DEG sets revealed several functional categories of significance. In the case of the F_up set, a strong enrichment of the cell wall category (10) was seen amongst upregulated genes compared with the genome as a whole, the DEG genes encoding proteins such as endoglucanase, polygalacturonases, pectate lyase, and xyloglucan endotransglucosylase/hydrolase. Although the significance of this observation is not clear, cell wall metabolism is known to play an important role in plant development as a whole [44]. In the M_up DEG set, the significant overrepresentation of genes associated with gluconeogenesis/glyoxylate cycle (category 06) presumably reflects the difference in metabolic demands of the two different types of inflorescence within the developmental window studied. The observed Male_up DEGs include genes encoding proteins such as citrate synthase and phosphoenolpyruvate carboxykinase, which are both known to accumulate strongly in sink tissues such as developing flowers [45,46].

In the case of the hormone metabolism category (17) found to be enriched in the M_up DEG set, a wealth of previous research has been conducted linking inflorescence development to this functional group. As a complement to our RNA-seq studies, we obtained biochemical data detailing the hormonal composition of the male and female inflorescences with respect to a range of different cytokinin and auxin forms. The strikingly larger quantities of t-ZOG and t-ZR found in the female samples point to a key role for these molecules in the sexual differentiation of the oil palm inflorescence. Cytokinins have been known to play an important role in numerous plant processes, including flowering, for many years [47]. An important early insight into the biological role of cytokinins in the regulation of plant morphology was obtained by Hirose et al. [48]. The latter authors demonstrated that overexpression of the rice cytokinin-inducible Type-A response regulator *OsRR6* resulted in changes to plant morphology, including that of the panicle. Ashikari et al. [49] showed that a QTL conferring increased grain productivity in rice, Gn1a, corresponded to the *OsCKX2* gene encoding a cytokinin oxidase/dehydrogenase that degrades cytokinin. Reduced expression of *OsCKX2* resulted in increased cytokinin accumulation in inflorescence meristems and the production of larger numbers of branches and flowers. In more recent studies on rice, Duan et al. [50] identified *CYTOKININ OXIDASE/DEHYDROGENASE 9* (*OsCKX9*) as a primary cytokinin response gene. Both CRISPR/Cas9-generated mutants of *OsCKX9* and transgenic plants overexpressing the same gene showed similar phenotypic changes, including a decrease in panicle size. These observations illustrate that the cytokinin signaling mechanism in which *OsCKX9* participates is subject to homeostatic control. A similar situation is likely to occur during the early differentiation of the oil palm inflorescence, for which we observed an enhanced accumulation of cytokinins in the female samples, accompanied by the upregulation of three different cytokinin hydroxylase genes involved in the degradation of this hormone. Recent studies have established a clear link between cytokinin signaling and the determination of inflorescence structure by *SPL* genes. Du et al. [51] observed that when expressed in rice, the maize *UB3* gene was able to affect panicle branching in two different manners: negatively when transgene expression was strong and positively when the transgene expression level was moderate. It was found that the UB3 protein could bind and regulate the promoters of *LONELY GUY* (*LOG*) and Type-A response regulator genes (ARRs), which act respectively in cytokinin biosynthesis and signaling. Again, evidence was obtained for the existence of a negative feedback loop enabling cytokinin homeostasis, but the overall results indicate that *UB3* and its rice orthology *IPA1* may regulate vegetative and reproductive branching by modulating cytokinin biosynthesis.

**Figure 5:**
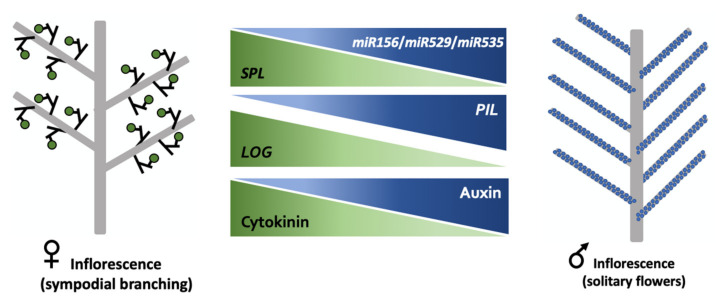
Possible regulatory interactions in oil palm inflorescence sexual differentiation.

In the case of oil palm, clear parallels can be noted between the present data and those discussed above. For our study, we included in our samples developmental stages from the appearance of floral bracts (male inflorescence) or floral triad bracts (female inflorescence) up to the initiation of floral meristems. Any gene expression or hormonal differences that distinguish the male and female inflorescences within this developmental window, especially in relation to branching, are therefore likely to reflect the difference between the determinate development of the male flowers and the initiation of new branching axes in the flower clusters of the female inflorescence. In this respect, our data fit the general pattern observed with rice, maize, and other species: higher branching activity is associated with increased expression levels of *SPL* genes, lower accumulation of their miRNA regulators, and higher cytokinin accumulation (Figure 5). The higher expression of genes encoding cytokinin-degrading enzymes in the female inflorescence presumably reflects the existence of a homeostatic feedback mechanism, as observed in the aforementioned studies by Duan et al. [50] Du et al. [51].

Further underlying complexity may also result from the involvement of other plant hormones. Our observation of opposing sex-dependent patterns of IAA and cytokinin accumulation in immature oil palm inflorescences is consistent with their generally observed antagonistic effects [52]. Auxins are well known to influence branching in different parts of the plant, often through mechanisms involving their differential transport [53]. In the context of inflorescence development, a recent study demonstrated that the rice auxin transporters OsPIN1c and OsPIN1d participate in the regulation of panicle development [54], and it can be noted that two different oil palm genes encoding auxin efflux carrier proteins displayed male-enhanced expression in this study. Other hormones such as strigolactones might also play underlying roles. Although no biochemical evidence was obtained for the involvement of the latter group in this study, their role in the determination of panicle architecture branching in rice via activation of the *OsCKX9* gene has been demonstrated [50], and in our dataset, the expression of an oil palm *DWARF14*-like gene encoding a probable strigolactone esterase [55] was observed to be considerably higher (approximately 8-fold) in the male inflorescence.

In conclusion, the early sexual differentiation of the oil palm inflorescence to produce male and female forms with different branching architectures is characterized by the differential expression of a number of key genes already known for their morphogenetic importance in other species, some of which are subject to miRNA-dependent post-translational regulation. Sexual differentiation is also characterized by contrasting profiles of accumulation of specific cytokinin and auxin forms, along with differential expression of genes involved in their metabolism and signaling pathways. Our study has allowed us to identify key elements involved in the underlying processes regulating this development. Future work should focus on a finer dissection of this complex system through developmental time course studies and spatial analysis of the patterns of gene expression and hormone signaling at a tissue-specific and cellular level. For the moment, tools for the investigation of gene functions in oil palm are limited and constrained by the large size and long life cycle of this plant [56]. Nevertheless, progress has been made in the development of transient and stable genetic transformation methods, including CRISPR-based technology [57], while heterologous expression in more experimentally amenable species such as rice [58] may also offer solutions for functional validation studies.

## 4. Materials and Methods

### 4.1. Plant Material

Immature inflorescences of oil palm (*Elaeis guineensis*) were harvested from 6 different individuals growing in the same experimental parcel at the Centre de Recherches Agricoles Plantes Pérennes station in Pobè, Bénin. Inflorescence samples A04M, A05F, A06F, and A07M were harvested from siblings of the same genetic cross, each named sample being taken from a single palm. In the same way, samples B04M and B05F were obtained from two additional palms that were siblings of a second genetic cross. Each studied palm was felled, then progressively dissected, removing the immature inflorescence present in the axil of each of its leaves. Inflorescences were referenced by leaf position with respect to the youngest emergent expanding leaf designated as leaf +1. The various stages of oil palm inflorescence and flower development and their corresponding leaf references are summarized in Figure S1. Because oil palm is a monoecious species producing distinct male and female inflorescences on the same plant, any individual sampled will usually contain a mixture of both inflorescence types, each corresponding to a different developmental stage. In our sampling, for each palm, inflorescences of the same sex occurring between leaves −3 and +16 were pooled. Depending on which sex predominated, either a male or a female sample was retained for analysis. By pooling inflorescences of the same sex from the same palm, it was feasible to carry out several different types of analysis on the same sample (small RNA, mRNA, hormone analysis), except in the case of samples B04M and B05F, for which insufficient material was obtained for hormone analysis. The developmental window targeted by our sampling started at the earliest stage for which inflorescence sex could be identified by hand lens examination and ended at early flower bud formation before reproductive organ differentiation had commenced (Figure S1). Samples were frozen immediately in liquid nitrogen and stored at −80 °C prior to analysis. Samples were homogenized by grinding with a mortar and pestle before being subdivided for separate analytical procedures. For illustrative purposes, representative samples of the inflorescence developmental stages of interest, harvested from other palms in the plantation, were processed for light microscopy by fixation, resin embedding, sectioning, staining, and visualized as described by [17].

### 4.2. Extraction of Small RNAs from Oil Palm Inflorescence and Analysis of miRNAs

Total RNAs (including small RNAs) were extracted using an RNeasy Plant Mini Kit with RLT and RWT buffers (Qiagen SAS, Courtaboeuf, France). DNase treatments were performed using the RNAeasy-free DNase set (Qiagen SAS, Courtaboeuf, France). After quantity and quality checks, purified small RNA sequencing was performed at MGX (Montpellier, France). Library preparation was carried out using an Illumina TruSeq Small RNA Kit Sample Prep Kit. Sequencing was performed on an Illumina HiSeq 2000 machine. Adapters and low-quality sequences were removed by means of CutAdapt [59], and trimmed reads ranging from 18 to 25 nucleotides in length were mapped to the *E. guineensis* reference genome (version EG5; https://www.ncbi.nlm.nih.gov/assembly/GCF_000442705.1/ accessed on 1 August 2021) using the MirDeep2 Mapper available on the University of Freiburg Galaxy Server (https://rna.usegalaxy.eu/ accessed on 1 August 2021) [60,61]. Read count and mapping statistics for small RNA-seq data are shown in Table S6. Individual small RNA sequences and their associated counts were extracted from the resulting collapsed reads file by text manipulation. For the purpose of miRNA annotation, a concatenated FASTQ file containing merged cleaned reads from all 6 samples was mapped. From the resulting collapsed reads file, annotation of oil palm *MIRNA* genes represented in the present dataset was performed by the sRNAanno platform at South China Agricultural University [21]. In order to study the accumulation of individual miRNAs across the 6 samples, their corresponding counts were normalized to give per million values (Table S2).

### 4.3. Extraction of Total RNAs and Analysis of Differential Gene Expression

Total RNAs were extracted from ground frozen inflorescence samples using a Plant RNA Mini Kit (Qiagen SAS, Courtaboeuf, France) following the manufacturer’s instructions. Synthesis of cDNA libraries and sequencing (using an Illumina NovaSeq 6000 sequencer) was carried out by Novogene (Cambridge, UK) for samples A05F, A07M, B04M and B05F. In the case of samples A04M and A06F, library preparation and sequencing (using an Illumina HiSeq 2000 sequencer) was carried out by Eurofins (Les Ulis, France). Reads obtained by Illumina sequencing were cleaned as before, then single-end mapped to the reference oil palm genome, using BWA-MEM on the University of Freiburg Galaxy Server [60]. Read counts were obtained from the resulting BAM files by means of the program Samtools-idxstats. Read count and mapping statistics for small RNA-seq data are shown in Table S7. The R-based software package DESeq2 [62] was used to study differential gene expression. A preliminary heatmap to visualize expression across all samples was obtained using the iDEP server [63] using the 50 most variable genes (Euclidean distance; average linkage; Z score cutoff 4; raw count input; centering of genes by mean subtraction; division by standard deviation to normalize samples). Subsequently, the DESeq package was used to identify genes displaying significantly different expression levels in the male and female inflorescences, applying the Padjusted cutoff < 0.05.

### 4.4. Functional Annotation of Genes and Identification of Enriched Categories

Functional annotation [64] of differentially expressed genes (DEGs) was carried out using the web-based Mercator/Mapman 3.6 tool [30]. In order to test for significant over-representation of a given functional category in each DEG set, Fisher’s exact test was employed, with a false discovery rate (FDR) cutoff of <0.05. Graphic illustrations were obtained using the method of [31]. Orthologues of DEGs in the model species *A. thaliana* and *O. sativa* were identified using the data published on the Plant Transcriptional Regulatory Map website (http://plantregmap.gao-lab.org accessed on 1 October 2021).

### 4.5. Analysis of Molecular Phylogeny

The molecular phylogeny of the three oil palm *SPL* genes was analyzed via their corresponding protein sequences by means of the online facility phylogeny.fr [65]. The 3 oil palm SPL sequences were compared with related sequences from rice and *A. thaliana* (clades III, V, VII, VIII and IX) identified by Preston and Hileman [23]. The following parameters were used: sequence alignment with MUSCLE 3.8.31; maximum likelihood tree implemented in PhyML program 3.1/3.0 aLRT; internal branch reliability assessed using the aLRT test (SH-Like); graphic representation of the phylogenetic tree with TreeDyn 198.3.

### 4.6. Analysis of Hormone Composition in Immature Oil Palm Inflorescences

Ground inflorescence tissues were freeze-dried prior to analysis of their auxin and cytokinin content (using 50 mg of dry powder per sample) at the NRC Plant Biotechnology Institute (NRC-PBI), Saskatoon, Canada. Ultraperformance liquid chromatography-ESI-tandem mass spectrometry, as described in detail by Chiwocha et al. [66], was used for this purpose. Graphs were produced using the R package ggpubr (https://rpkgs.datanovia.com/ggpubr/ accessed on 1 December 2021), and statistical tests (Wilcox method) were carried out using the package rstatix (https://rpkgs.datanovia.com/rstatix/ accessed on 1 December 2021).

## Figures and Tables

**Table 1:** Micro-RNAs with potential targets represented in the gene sets showing sex-dependent expression. Expression values were normalised using DESeq for DEGs and per million counts for miRNAs prior to the calculation of M/F ratios. Only miRNAs with an expression ratio above 2 or below 0.5 were considered. Pale blue highlighting in the left-hand column indicates miRNAs for which target mRNAs with a reverse accumulation profile were identified. The “∞” symbol indicates that miR156 was detected only in the male inflorescence, albeit in small quantities.

miRNA_ID	Sequence	M/F Ratio of miRNA	Type of Target	DEG mRNA Target	M/F Ratio of DEGs
***miR156d***	TGACAGAAGAGAGTGAGCACC	∞	SBP	*EgSPL14-1* (LOC105050210) *EgSPL14-2* (LOC105061255) *EgSPL143-3* (LOC105050485)	0.1806 0.3275 0.4332
***miR529a***	AGAAGAGAGAGAGTACAGCCT	8.2980	SBP	*EgSPL14*-1 (LOC105050210) *EgSPL14-2* (LOC105061255) *EgSPL14-3* (LOC105050485)	0.1806 0.3275 0.4332
***miR156a***	CTGACAGAAGAGAGTGAGCAC	8.1495	SBP	*EgSPL14*-1 (LOC105050210) *EgSPL14-2* (LOC105061255) *EgSPL14-3* (LOC105050485)	0.1806 0.3275 0.4332
***miR535a***	TGACAACGAGAGAGAGCACGC	7.2660	SBP	*EgSPL14*-1 (LOC105050210) *EgSPL14-2* (LOC105061255) *EgSPL14-3* (LOC105050485)	0.1806 0.3275 0.4332
***miR156b***	TTGACAGAAGATAGAGAGCAC	7.2342	SBP	*EgSPL14*-1 (LOC105050210) *EgSPL14-2* (LOC105061255) *EgSPL14-3* (LOC105050485)	0.1806 0.3275 0.4332
***miR5179***	TTTTGCTCAAGACCGCGCAAC	4.3972	DEF	*EgDEF1* (ID105033334)	0.0307
***miR167b***	TGAAGCTGCCAGCATGATCTA	3.5564	ARF	LOC105034684 LOC105055363	0.3410 0.3620
***miR167a***	TGAAGCTGCCAGCATGATCT	3.2110	ARF	LOC105055363	0.3620
***miR164a***	TGGAGAAGCAGGGCACGTGCA	2.6633	NAC	LOC105058561 LOC105056634	3.3287 22.0167
***miR397b***	TCATTGAGTGCAGCGTTGATG	2.4699	Laccase	LOC105036954	14.0474
***miR397a***	TCATCGAGTGCAGCGTTGATG	0.4993	Laccase	LOC105036954	14.0474

## Data Availability

The data presented in this study are available on request from the corresponding author. The data are not publicly available at the time of publication due to short term privacy considerations.

## Supplementary Materials

The following supporting information can be downloaded at: https://www.mdpi.com/article/10.3390/plants11050685/s1, Figure S1: Time course of oil palm inflorescence development, Figure S2: Heatmap of differential gene expression, Figure S3: Molecular phylogeny of proteins encoded by the differentially expressed oil palm SPL genes; Table S1: List of miRNA genes identified, Table S2: Accumulation profiles of mature miRNAs, Table S3: List of differentially expressed genes (DEGs), Table S4: List of DEGs in each identified functional category, Table S5: Summary of gene enrichment data, Table S6: Hormone analysis data, Table S7: Illumina/mapping summary—small RNA and mRNA.
