# Supplementary material for: Micro-RNA-Regulated SQUAMOSA-PROMOTER BINDING PROTEIN-LIKE (SPL) Gene Expression and Cytokinin Accumulation Distinguish Early-Developing Male and Female Inflorescences in Oil Palm (Elaeis guineensis)
Source: Plants (Basel). 2022 Mar 2;11(5):685. doi: 10.3390/plants11050685 (PMC8912876; doi:10.3390/plants11050685)
Supplement: Supplementary file 1 [file plants-11-00685-s001.zip › Supplementary/Supp data legends.pdf]

Figure S1. Schematic description of inflorescence development in oil palm. Leaf positions, relative to the youngest expanding leaf (L+1), are shown in red. The shaded orange box denotes the developmental stages harvested for the purposes of this study.

Figure S2. Heat map depicting the expression of the 50 oil palm genes displaying the most variable expression across all eight samples. An analysis was performed using the web-based application iDEP (Ge et al., 2018), with data centering by subtraction of the average expression level for each gene in combination with a distance matrix 1- r, where r is Pearson's correlation coefficient. Log_2 values produced the colors indicated on the heatscale. Clustering revealed that male samples (labelled in blue) and female samples (labelled in yellow) grouped together in distinct groups. *Arabidopsis thaliana* orthologs of the oil palm genes, where identified (http://planttfdb.gao-lab.org/), are indicated to the right of the oil palm locus numbers.

Figure S3. Molecular phylogeny of the three oil palm SPL protein sequences. An analysis was performed by means of the online facility phylogeny.fr [66]. The 3 oil palm SPL sequences were compared with related sequences from rice and *A. thaliana* (clades III, V, VII, VIII and IX) as identified by Preston and Hileman [23]. Parameters used are specified in the Materials and Methods section.

Table S1. Annotation of the identified oil palm *MIRNA* genes. Identification of *MIRNA* genes represented in the present dataset was performed by the sRNAanno platform at South China Agricultural University [21]. In the table are shown the sequences of primary transcripts along with mature and star forms of the miRNA, with specification of the strand concerned and the genomic location of the identified gene. Genes already catalogued in the sRNAanno database are indicated. Symbols in column "other details" indicate the following characteristics: •, minor shift in nucleotide position(s) compared to earlier annotation; §, inversion of mature and star strands compared to earlier accession. Other abbreviation: N/A, not applicable.

Table S2: Accumulation profiles of mature miRNAs. The accumulation profile across the 3 male and 3 female inflorescence samples of each identified mature miRNA form, expressed as normalised per million values, is shown along with their corresponding target mRNA types (in the case of known miRNA families) or potential mRNA targets (identified using the psRNATarget server in the case of unclassified miRNAs [22].For miRNAs displaying a M/F accumulation ratio above 2 or below 0.5, the presence or otherwise of potential mRNA targets amongst the DEGs is indicated. In cases where the miRNA and putative mRNA target show reversed M/F accumulation profiles, green shading is used in the right hand column.

Table S3: List of differentially expressed genes (DEGs). The DESeq2 package, applying a padjust threshold of 0.05, allowed the identification of 286 differentially expressed genes (DEGs) displaying higher transcript accumulation in the male inflorescence ("M_up" category) and 636 genes that were more highly expressed in the female inflorescence ("F_up" category). In the table shown, DEGs are ranked by their log2fold change values. Normalized expression values calculated for each sample using DESeq2 are shown, as are information on the oil palm genes and their orthologs, where identified, in the model species *A. thaliana* and *O. sativa*.

Table S4: List of DEGs by functional category. DEGs are indicated by their locus ID, along with their corresponding log2foldchange values and gene descriptions (also shown in Table S3).

Table S5: Gene enrichment data. Gene functions were attributed according to Lohse et al. (2014) and percentages of genes belonging to each category compared with the entire palm gene dataset as described in the Materials and Methods section. Significant gene enrichment was tested for using Fisher's exact test with FDR (fold discovery rate) cutoff < 0.05. The same data shown are represented graphically in Figure 3. Significant cases of enrichment are highlighted in green for the M_up DEG set and blue for the F_up DEG set.

Table S6: Hormone analysis data. We analysed the auxin (3 forms) and cytokinin (7 forms) contents of samples A04M, A05F, A06F and A07M using the same tissue samples as were used for the transcriptomic analyses. Measurements are expressed as ng/g dry weight. Mean and standard deviation values are shown. Abbreviations: IAA; indole-3-acetic acid; IAA-Asp, indole-3-acetyl-aspartate; IAA-Glu, in-dole-3-acetyl-glutamate; c-ZOG, cis-zeatin O-glucoside; c-ZR, cis-zeatin riboside; dhZR, dihydrozeatin riboside; iPA, isopentenyladenine; t-Z, trans-zeatin; t-ZOG, trans-zeatin O-glucoside; t-ZR, trans-zeatin riboside. The same data are represented in Figure 4 with significance calculations.

Table S7: Summary of Illumina sequencing and mapping data for small RNA and mRNA studies. Statistics are shown for raw reads, cleaned reads, total collapsed reads (for small RNA-seq data) and mapped reads (for mRNA studies).
