# Supplementary figures and images for: Micro-RNA-Regulated SQUAMOSA-PROMOTER BINDING PROTEIN-LIKE (SPL) Gene Expression and Cytokinin Accumulation Distinguish Early-Developing Male and Female Inflorescences in Oil Palm (Elaeis guineensis)
Source: Plants (Basel). 2022 Mar 2;11(5):685. doi: 10.3390/plants11050685 (PMC8912876; doi:10.3390/plants11050685)

**VEGETATIVE  
MERISTEM**

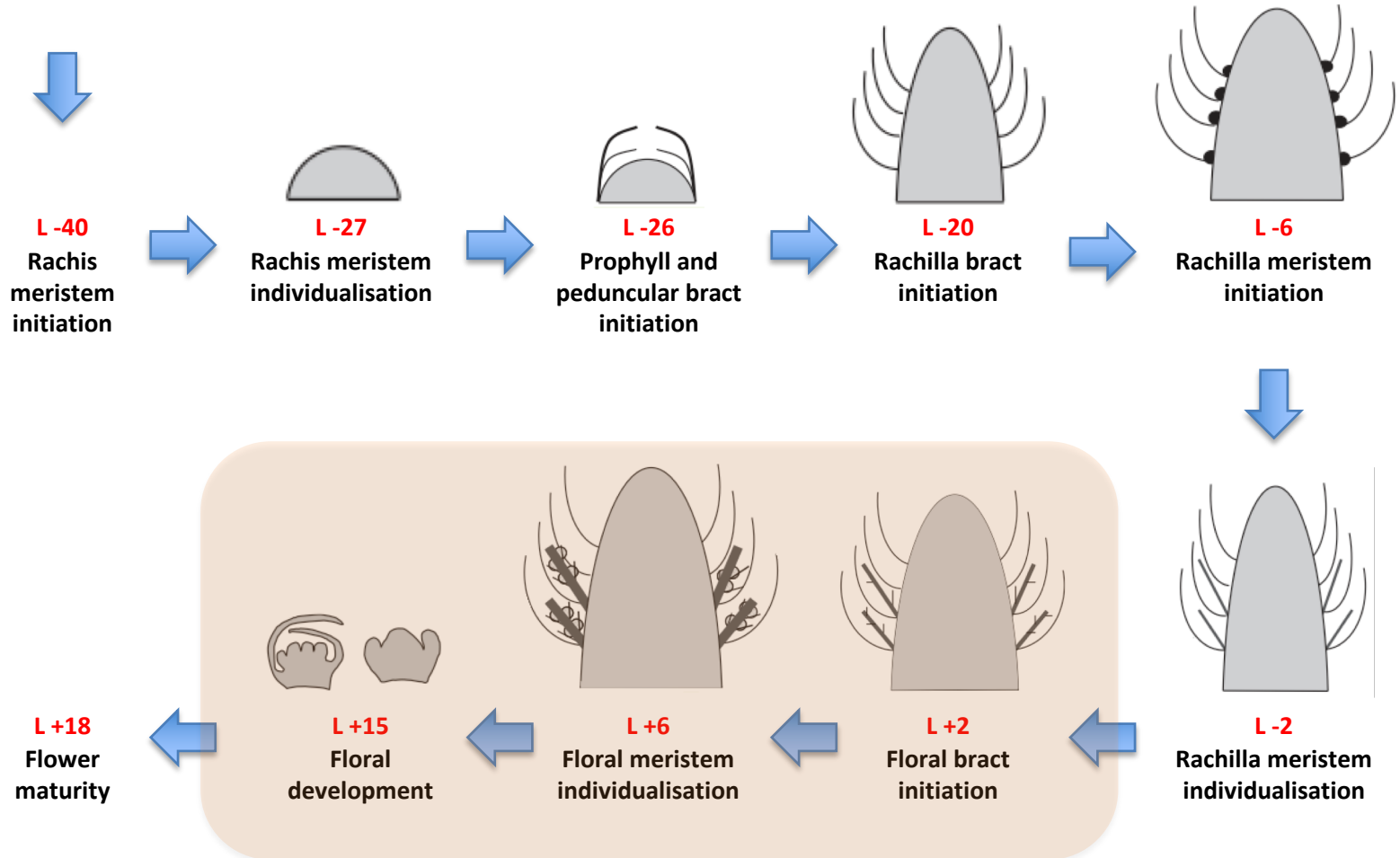

Supplement: Supplementary file 1 [file plants-11-00685-s001.zip › Supplementary/Figure S1.pdf]

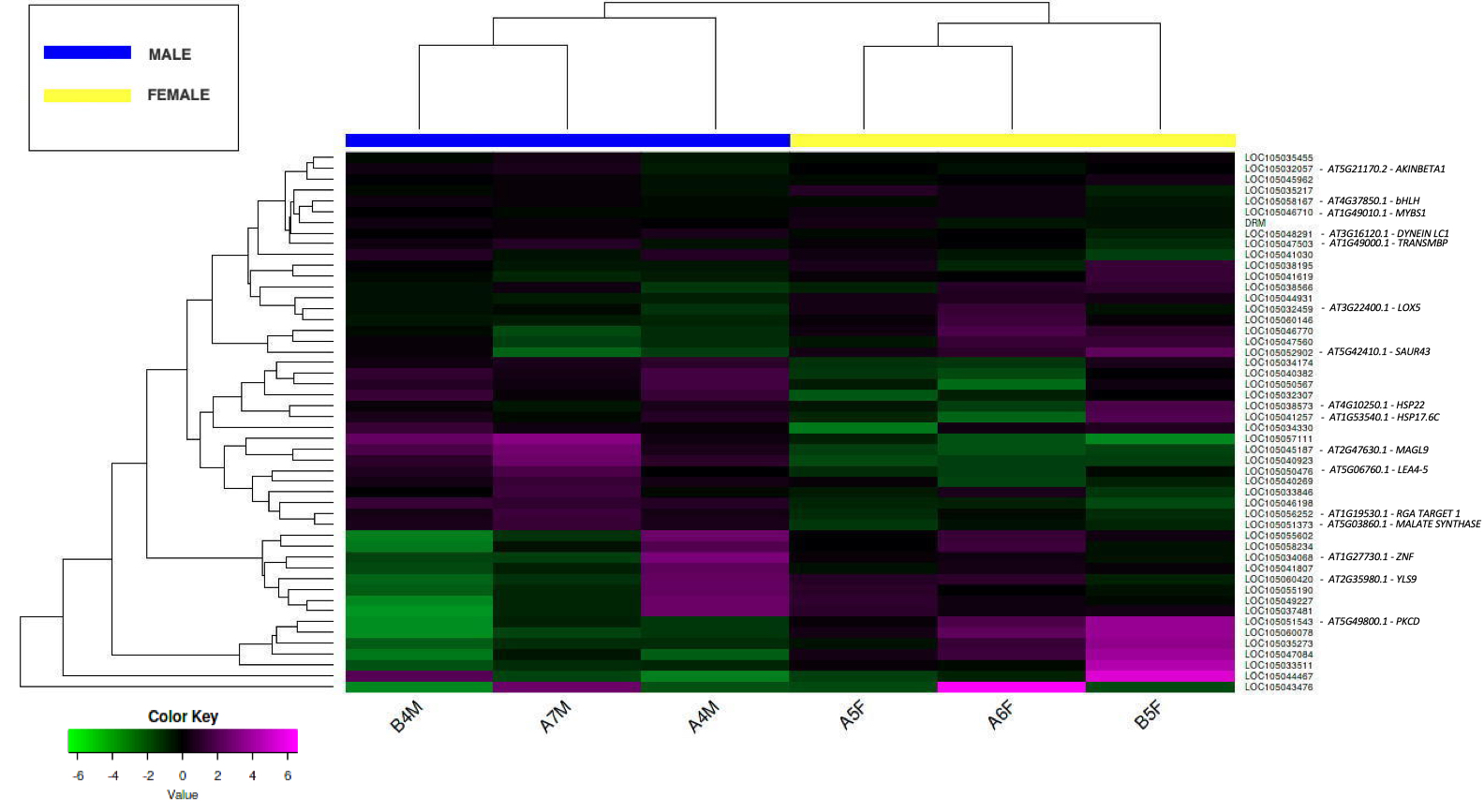

Supplement: Supplementary file 1 [file plants-11-00685-s001.zip › Supplementary/Figure S2.jpg]

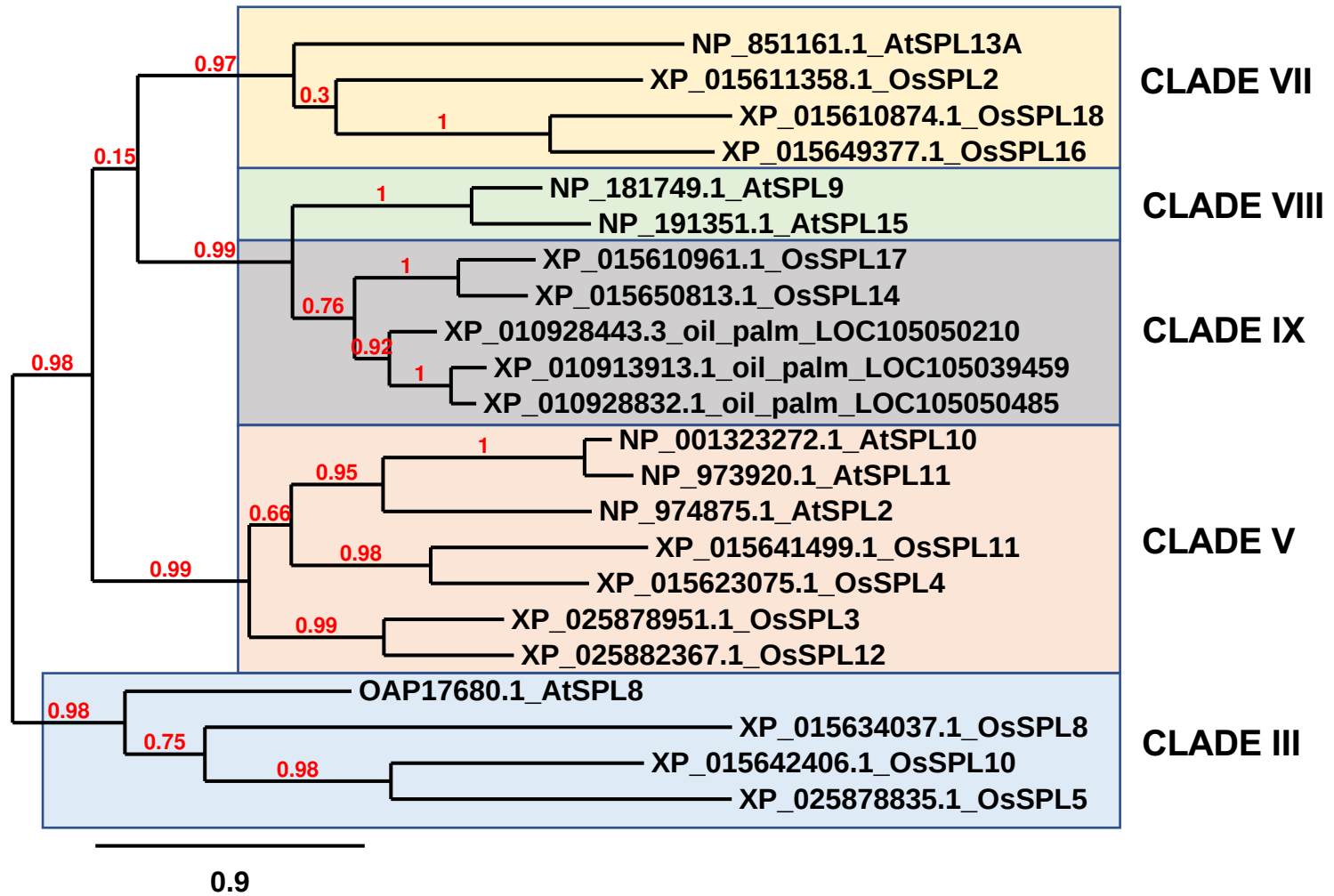

Supplement: Supplementary file 1 [file plants-11-00685-s001.zip › Supplementary/Figure S3.pdf]
